# *Streptococcus bovis* Hip and Knee Periprosthetic Joint Infections: A Series of 9 Cases

**DOI:** 10.7150/jbji.36923

**Published:** 2020-01-01

**Authors:** Jeremy C. Thompson, Ashton H. Goldman, Aaron J. Tande, Douglas R. Osmon, Rafael J. Sierra

**Affiliations:** 1 Department of Orthopedic Surgery; 2 Division of Infectious Diseases, Department of Internal Medicine, Mayo Clinic, Rochester, MN.

## Abstract

**Introduction:** Prosthetic joint infection (PJI) due to *Streptococcus bovis* group (SBG), specifically* S. bovis* biotype I (*S. gallolyticus*), is rare and associated with colorectal carcinoma. Little has been published regarding *SBG* PJI. We analyzed nine cases of SBG PJI at our institution, the largest series to date.

**Methods**: The medical records of patients diagnosed with SBG PJI between 2000-2017 were reviewed. Patients were followed until death, failure, or loss to follow-up. Mean follow-up was 37 months (range 0.5-74 months).

**Results:** Nine PJI in 8 patients with mean prosthesis age at diagnosis of 8 years (range 4 weeks-17 years) were identified. The median duration between symptom onset and treatment was 38 weeks (range 0.3 weeks-175 weeks). 8/9 had their PJI eradicated with treatment based on acuity of symptoms. Acute PJI (2) was treated with DAIR, and chronic PJI (7) was treated with 2-stage revision arthroplasty. 1 PJI with chronic PJI developed recurrent infection after initial treatment. All patients received post-operative IV antibiotics. 7/8 patients received Ceftriaxone. Three patients received lifelong oral antibiotics. 7/8 patients underwent colonoscopy. 5/7 patients were found to have polyps following PJI diagnosis with one carcinoma and two dysplastic polyps. The two patients without polyps had identifiable gastrointestinal (GI) mucosal abnormality: tooth extraction prior to symptom onset and diverticulosis on chronic anticoagulation.

**Conclusion:** SBG PJI is typically due to hematologic seeding. Colonoscopy should be pursued for patients with SBG PJI. Surgical treatment dictated by infection acuity and 6-week course of Ceftriaxone seems sufficient to control infection.

## Introduction

Periprosthetic joint infection (PJI) is a major complication with an incidence of 0.3% to 4% after primary total hip arthroplasty (THA) and 0.4% to 3.9% for primary total knee arthroplasty (TKA) 1-6. Several risk factors for the development of PJIs are well-known and include an immunocompromised state, diabetes mellitus, poor nutrition, and wound complications, among others 3-5, 7. The most common PJI pathogens are *Staphylococcus aureus* and coagulase-negative staphylococci; however, there are other less common PJI-causing bacteria that have specific treatment, diagnostic, and prognostic implications 8, 9.

The *Streptococcus bovis* group (SBG) is an uncommon cause of PJI. Previous reports have shown a strong association between SBG bacteremia and concomitant colorectal carcinoma 10, 11. With the development of more refined identification methods, the nomenclature and disease associations of SBG have been updated to show association between specifically *S. bovis* biotype I (*S. gallolyticus subsp. gallolyticus*) and colorectal cancer 12-14. However, little has been published regarding SBG septic arthritis, and even less has been reported regarding SBG PJI 15-20. The purpose of this paper is to report one institution's experience with SBG PJI and answer the following questions: 1. what is the proportion of SBG PJI to all PJI, (2) what is the relationship between SBG PJI and any potential sources including colonic/rectal malignancy, and (3) what are the outcomes of surgically treated SBG PJIs.

## Methods

After IRB approval (IRB # 18-000453, 2/8/2018), we identified all cases of SBG PJI from our institutional PJI registry that includes all hip and knee PJI diagnosed and/or treated at our institution. Cases of SBG between January 1, 2000 and May 1, 2017 were identified. All patients over 18 years of age who underwent initial surgical management of a SBG PJI at our institution were included. Detailed patient information was abstracted from the medical record. SBG PJI was diagnosed based on Musculoskeletal Infection Society (MSIS) criteria and retrospectively confirmed using the proposed 2018 MSIS criteria 21, 22. Medical and surgical therapies were not standardized and were performed at the discretion of the treating physicians. Patients were followed after treatment for recurrence of infection or death. Treatment failure was determined using previously published criteria developed through the Delphi process 23. Acute PJ I was defined as infectious symptom duration less than 3 weeks or diagnosis and/or symptom onset within 4 weeks from arthroplasty. Chronic PJ I was defined as infectious symptoms during a period greater than what is noted above. The data are reported using standard summary statistics, including means for continuous variables, and counts and percentages for categorical variables. Descriptive statistics were analysed using JMP, Version 9.0.1 (SAS Institute Inc.).

## Results

There was a total of 2,459 PJIs in 1,930 patients during the study period treated at our institution. The nine cases of SBG PJI accounted for 0.4% of the total PJI at our institution over the 18-year study period. Nine SBG PJI (8 patients) were included in our patient cohort. The biotype of SBG was known in one patient (*S. gallolyticus*). One patient had simultaneous bilateral PJI. The average age at time of PJI diagnosis was 75 years old (range 65 - 95 years), and the average BMI was 32 kg/m^2^ (range 25 - 35 kg/m^2^ (Table 1). All patients obtained blood cultures at time of diagnosis of PJI, none of which returned positive. No patient received antibiotics within 3 months of obtaining blood or joint fluid culture. 2 of 8 patients underwent echocardiogram after PJI diagnosis with 1 patient showing signs of endocarditis. This patient had a known history of native valve SBG endocarditis treated with a 6 week courses of ceftriaxone and ertapenem followed by porcine valve replacement after resolution of endocarditis 11 months after diagnosis. He developed simultaneous bilateral knee *S. gallolyticus* PJI 6 months after heart valve replacement.

Seven of the 8 patients underwent colonoscopy with biopsy after diagnosis of SBG PJI with one patient deferring colonoscopy due to comfort care management. Of the 7 patients undergoing colonoscopy, 1 patient was diagnosed with colorectal carcinoma, 1 patient was found to have high grade dysplastic polyps, 1 patient had low-grade dysplastic polyps, and 2 patients were found to have adenomatous polyps without dysplasia (Table 2). Therefore 5 of 7 patients had colorectal processes at the time of the SBG PJI, while 2 patients had no evidence of polyps or carcinoma on colonoscopy**.** The 2 patients without GI tract neoplasm identified on colonoscopy had other mucosal abnormalities: one patient underwent tooth extraction 5 weeks prior to onset of symptoms, and the other patient was found to have diverticulosis, on chronic anticoagulation for atrial fibrillation, a history of a GI bleed, and had a history of bariatric surgery. Within the subset of patients with colonic neoplasm, multiple patients were found to have additional sources of GI mucosal abnormality and/or bacteraemia. These abnormalities include diverticulosis (3), history of GI bleed (3), prior bariatric surgery (2), liver hemangioma (1), arteriovenous malformation (1), candida esophagitis (1), and history of SBG endocarditis and subsequent porcine cardiac valve replacement.

The mean age of the prosthesis before surgical treatment of PJI was 8 years (4 weeks - 17 years). Two acute haematogenous PJIs were treated with irrigation, debridement, and polyethylene exchange (DAIR). The mean duration of symptoms before DAIR was 3.5 days. The seven remaining PJI presenting as chronic infection were treated with a 2-stage revision with plans for subsequent reimplantation (Table 3). The mean duration of symptoms before 2-stage surgical treatment was 38 weeks (5 weeks - 175 weeks). One patient (with simultaneous bilateral knee PJI) elected to have his reimplantation performed closer to home and was subsequently lost to follow- up. Four of the remaining 5 PJI subsequently went on to reimplantation while 1 patient opted to keep the articulating spacer as it was functioning well and had no evidence of reinfection. The mean time to re- implantation was 48 weeks (range 10 - 109 weeks). All patients received IV antibiotics, with the treatment details presented in Table 2. Ceftriaxone (6-week course) was the most commonly used IV antibiotic with only 1 patient using an alternative (ertapenem due to side effects). Three total patients were recommended lifelong antibiotic suppression. The two patient receiving DAIR treatment for acute PJI and the one patient with chronic PJI deemed a treatment failure were given lifelong oral antibiotic suppression at the discretion of the treating physicians.

Of the 7 patients who underwent planned 2-stage revision arthroplasty, one joint sustained a subsequent infection with another organism. This patient was deemed a treatment failure. She required one return trip to OR for irrigation & debridement two separate return trips to OR for revision spacer placement before definitive re-implantation. This patient presented as a 69-year-old female with past history of prior bariatric surgery with resultant malnutrition/hypoalbuminemia, obesity, and diabetes mellitus that underwent primary right TKA for continued pain after non-operative management of patella fracture and primary knee osteoarthritis. Her initial surgery was complicated by postoperative arthrofibrosis for which she underwent manipulation under anaesthesia at 2 months postoperatively. She continued to have chronic right knee pain and underwent revision TKA for loosening one year later. While she did have a positive bone scan before this revision TKA, cultures were not obtained at that time. She reported recurrence of knee pain after a 6-month pain free period. She managed this pain conservatively for 3.5 years before presenting to Mayo Clinic. Aspiration of the knee was then obtained which revealed purulent fluid, cell count of 15,390, and 97% polynuclear cells. Cultures returned as pan-sensitive *S. bovis*. Further speciation was not available. Due to its association with colorectal cancer, she underwent colonoscopy which returned with multiple tubulevillous polyps and associated low-grade dysplasia. She subsequently underwent placement of antibiotic spacer and was started on IV ertapenem (ceftriaxone deferred due to gastric irritability) and oral TMP-SMX. She subsequently developed right knee drainage and sinus tract formation for which she returned the operating room at 2 weeks post-op for I&D and sinus tract excision. She was maintained on IV ertapenem, and she was placed in a long leg cast after this procedure. Tissue cultures at this time were negative for causal organism. Unfortunately, 1 month from this I&D, she presented with recurrent wound drainage. Serum ESR and CRP at this time were 45 mm/1^st^ hr and 21 mg/dL respectively. Aspiration revealed 2,588 nucleated cells and 47% neutrophils. The decision was made to return to the operating room for exchange of antibiotic cement spacer and antibiotics were switched to ceftriaxone for 10 weeks. 3 months after this surgery, she again presented with a persistently draining anterior right knee. The decision was made for repeat antibiotic cement spacer placement with assistance from plastic surgery for pedicled gastrocnemius flap coverage and split thickness skin graft for wound coverage. Tissue cultures at this time were negative for causal organism. IV antibiotics were changed to piperacillin-tazobactam for 3 months after surgery. At a follow-up visit 6 weeks from this surgery, she again was found to have a draining, non-healing right knee wound. She again returned to the operating room for exchange of her antibiotic cement spacer. Tissue cultures at this time were positive for ticarcillin-clavulanate and ceftazidime-resistant* Stenotrophomonas Maltophilia*. She was thus given the following antibiotics simultaneously over a 4-week period: 7-day course of IV daptomycin, 4-week course of ertapenem, and 2-week course of oral levofloxacin followed by 2-week course of TMP-SMX. She denied pain post-operatively but maintained mildly elevated inflammatory markers. An indium scan was performed which returned negative for signs of infection. Because she was doing well clinically off of antibiotics, the decision was made to perform right knee reimplantation with hinged prosthesis 81.1 weeks after prior cement exchange (108.9 weeks from 1^st^ spacer placement). Before re-implantation, she had a repeat colonoscopy which identified an arteriovenous malformation (AVM) in the ascending colon as well as multiple pedunculated adenomatous polyps without dysplasia. She has been on lifetime cefadroxil since this operation. Inflammatory markers have since normalized and repeat aspiration was within normal limits at last follow-up 21 months after her last surgery.

## Discussion

While SBG*,* specifically* S. Bovis* biotype I *(S. gallolyticus)* infection such as bacteremia and/or infective endocarditis have been shown to be correlated with colorectal cancer, little has been published regarding *S. bovis* PJI. In our review, SBG was responsible for 0.4% of all PJI at our institution over the study period. In our study of 9 cases of SBG PJI, we found one case of colorectal cancer and 2 cases of dysplastic polyps. Additionally, all 8 patients were found to have at least one GI mucosal abnormality predisposing to bacterial translocation to the bloodstream.

Colorectal cancer is currently the fourth most common cancer in the United States. While the 5-year survival rate of those with colorectal cancer is only 65%, it has been shown that early treatment of polyps, diagnosis, and definitive treatment has a positive impact of survival 24, 25*.* The *SBG* group are members of the Group D streptococcus family and are Gram-positive cocci that can be part of the normal GI flora in 5-16% of adults 10, 11. Furthermore, it has been shown that colorectal carcinoma alters the native gut microbiosis which promotes *Streptococcus gallolyticus* proliferation^26^. It has been suggested that this bacterium releases inflammatory proteins which stimulate chronic inflammation, inhibit apoptosis, and increase angiogenesis which may lead to neoplasm formation 27, 28. Therefore SBG PJI may play a role in the early diagnosis of colorectal cancer.

Prior to this case series, the authors were able to identify only 6 case reports of SBG PJI 1, 2, 8, 15-20, 29. Four of the reported 6 cases had concomitant colorectal cancer, while the other cases had endocarditis or had premalignant polyps 15-20. One patient was found to have colorectal cancer, and 2 patients were found to have premalignant polyps. In other disease states, the association between colorectal cancer and *SBG* infection appears to be most closely associated with the SBG biotype I (*S. gallolyticus* subspecies *gallolyticus*) 13. Subspeciation was identified in only 1 previous case report (*S. gallolyticus*) and this patient was found to have associated colonic malignancy^18^. In our study, the subspecies or SBG biotype causing infection for our patients was unknown in all but 1 patient (bilateral knee PJI) who was found to have *S. gallolyticus* PJI. Despite this lack of subspecies information and despite our lower rate of diagnosis of definitive colonic malignancy than previously published, there does still appear to be a correlation between GI mucosal abnormalities and SBG PJI.

There was one patient with SBG PJI requiring return to the operating room for spacer exchange prior to reimplantation due to a recalcitrant infection. It should be noted that this patient received a dissimilar antibiotic and has multiple risk factors for PJI as noted above. She has since been reimplanted and is on life-long suppression. Seven of 8 patients with available follow-up had their PJI eradicated with DAIR or 2-stage treatment depending on the acuity of their PJI. Acute PJI was treated with DAIR, and chronic PJI was treated with 2-stage revision arthroplasty. The 2 patients with acute PJI treated with DAIR received lifelong antibiotic suppression due to one patient deferring prolonged IV antibiotics due to comfort care management and the other patient due to a history of multiple revisions. This suggests that *S.bovis* PJI can be treated successfully with DAIR vs. 2-stage treatment and a 6-week course of appropriate IV antibiotics with additional antibiotics at the discretion of the treating physicians.

The major limitations to our study are inherent to with observational, retrospective nature. The small sample size is a function of the low incidence of disease. However, our institutional PJI registry has allowed us to retrospectively capture these cases for review and create the largest case series to date. Since our data was collected from a single tertiary referral medical center, there is potential for referral bias. This also contributed to a high variation in follow-up as multiple patients elected to follow-up at their respective outside facilities. Also, as previously mentioned, full speciation of the SBG subspecies was not obtained and/or recorded for all but 1 of the patients in the series. Information regarding the specific SBG subspecies would no doubt add to the association of colorectal cancer, GI mucosal abnormality, and PJI.

After review and analysis of our patient cohort, SBG PJI is relatively uncommon and results from late hematogenous seeding. As the majority of cases showed sensitivity to Ceftriaxone, surgical treatment dictated by infection acuity and medical treatment consisting of a 6-week course of Ceftriaxone seems sufficient to control infection. Risk factors for GI mucosal breakdown, including colorectal cancer, could be identified for those patients that elected to undergo colonoscopy. This data supports obtaining a colonoscopy in patients diagnosed with SBG PJI. This study provides insight into one potential mechanism of late hematogenous PJI.

## Figures and Tables

**Table 1:** Demographic data related to 9 cases of SBG PJI.

PJI #	Sex	Age at time of PJI	BMI	Affected Joint	Co-Morbidities
1	Female	65	32	Left Hip	None
2	Female	95	Unknown	Right Knee	diabetes mellitus type 2, chronic kidney disease, hypertension, congestive heart failure, coronary artery disease
3	Male	75	34	Left Hip	hypertension, coronary artery disease, peripheral vascular disease
4	Male	72	30	Left Hip	Atrial fibrillation (Coumadin), EtOH use, Prior bariatric surgery, previous GI bleed, diverticulosis
5	Female	79	31	Right Knee	None
6	Male	70	35	*Left* Knee	Prior *S. gallolyticus* native valve endocarditis, diabetes mellitus type 2, Prior venous thromboembolism (Coumadin), Tobacco use, chronic kidney disease, peripheral vascular disease, coronary artery disease, Prior Diskitis
7	"	"	"	*Right* Knee	"
8	Female	69	31	Right Knee	History of bariatric surgery, diabetes mellitus type 2, Hx venous thromboembolism, iron-deficient anemia, Hypothyroidism
9	Female	81	25	Left Hip	coronary artery disease, atrial fibrillation (pacemaker), congestive heart failure, Depression, Liver hemangiomas, gastrointestinal reflux disease
Mean (Range)	37.5% Male	75 (65 - 95)	32 (23 - 35)	44.4% Hips	-----

**Table 2:** Treatment Outcomes, Colonoscopy data, and GI mucosal risk factors related to SBG PJI in the current cohort.

Patient #	Symptom Duration	Surgical Management	Antimicrobial Therapy	Return to OR?	Outcome	Follow- up Duration	Colonoscopy Results	GI Comorbidities/ Findings	Other
1	44 weeks	2-Stage	IV Ceftriaxone (6 weeks) `	No	Reimplantation at 55 weeks	74 months	Tubulovillous Polyps - High grade dysplasia	None	Diagnosed with ovarian cancer after resolution from PJI
2	1 week	Poly Exchange	IV Ceftriaxone (2 weeks); Oral Cephalexin (indefinite)	No	Death (unknown cause)	0.5 months	Deferred	Deferred	Comfort care. Cause of death unknown
3	12 weeks	2-Stage	IV Ceftriaxone (6 weeks)	No	Reimplantation at 10 weeks	68 months	Colorectal carcinoma (T1N0M0)	Diverticulosis	N/A
4	2 days	Poly Exchange	IV Ceftriaxone (6 weeks); Oral Penicillin VK (indefinite)	No	Clear of infection and downtrending ESR/CRP	51 months	Indeterminate* (*Poor prep; also CT found indeterminate)	Diverticulosis; Prior bariatric surgery	Pre-operative inguinal hernia s/p repair
5	5 weeks	2-Stage	IV Ceftriaxone (6 weeks)	No	Delayed wound closure; reimplantation at 10 weeks	44 months	Clean	None	Tooth extraction w/ Amoxicillin ppx 5wk prior to onset; Delayed wound healing without infection - Tx w/ wet/dry dressing
6*	10 weeks	2-Stage	IV Ceftriaxone (6 weeks)	No	Reimplantation at OSH; Clear of infxn at last follow-up prior to reimplantation (13 months)	13 months	Adenomatous polyp, Candida Esophagitis	Diverticulosis; Candida esophagitis	Pre-operative endocarditis; Smoker (1PPD); History of GI bleed
7*	10 weeks	2-Stage	"	No	"	"	"	"	"
8	175 weeks	2-Stage	IV ertepenem; Cefadroxil (indefinite)	Yes	(1) Superficial I&D, (2-3) Revision abx cement spacer x 2 prior to final reimplatntation (109 weeks after dx); Clear of infection at last f/u	46 months	Tubulovillous Polyp (low grade dysplasia); AVM	Prior bariatric surgery	History of rectal bleed (anticoagulation)
9	11 weeks	2-Stage	IV ceftriaxone (6 weeks)	No	Reimplantation deferred (functioning spacer)	22 months	Adenomatous Polyp, Diverticulosis	Diverticulosis; Liver hemangiomas, History of choledocolithiasis	Chronic anticoagulation and history of GI bleeds
	38 weeks (2 days - 175 wks)	78% with 2-Stage	89% with IV Ceftriaxone 89% with ≥ 6 weeks IV Tx 33% with chronic suppression	11% failure (1 of 9)	Time to reimplantation 48 weeks (range 10 - 109 weeks).	37 months (0.5 - 74)	14% colorectal cancer (1 of 7) 29% dysplastic Polyps (2 of 7) 14% adenomatous polyps only (1 of 7)		

**Table 3:** Infection data of the current series.

Patient #	Prosthetic Joint (Revisions)	Revision Surgeries	Clinical Presentation	Microbiology	Aspirate (Cells, % PMN)	Age of Prosthesis at time of infection
1	Left Hip	Revised x 3 (wear), Revised x 1 (loosening)	Pain, fever, erythema, sinus tract x 44 weeks	Aspirate: (+) SBG -- Tissue: (+) SBG, S. virdans	10,625 cells, 98% PMN	2 years
2	Right Knee	None	Chest pain, shortness of breath, knee pain x 1 weeks	Aspirate: (+) SBG	27,612 cells, 85% PMN	Unknown (unknown date of primary)
3	Left Hip	Revised x 1 (wear); Revised x 2 (loosening)	Pain x 12 weeks	Aspirate & Tissue: (+) SBG	28,281 cells, 97% PMN	4 years
4	Left Hip	Revision x 1 (progression of Hemi OA); Revision x 1 (periprosthetic fracture)	Pain, Fever x 2 days	Aspirate & Tissue: (+) SBG	64,001 cells, 97% PMN	4 weeks
5	Right Knee	None	Pain, loosening x 5 weeks	Aspirate & Tissue: (+) SBG	98,164 cells, 97% PMN	17 years
6*	Left Knee	None	Pain, Sinus tract x 10 weeks	Aspirate: (+) SBG -- Tissue (-)	213,576 cells, 97% PMN	12 years
7*	Right Knee	None	Pain x 10 weeks	Aspirate: (+) SBG -- Tissue (+) P acnes	321,380 cells, 98% PMN	12 years
8	Right Knee	Revision x 1 (loosening)	Pain, Swelling x 175 weeks	Aspirate: (+) SBG -- Tissue (-)	15,390 cells, 97% PMN	1 yr
9	Left Hip	None	Pain x 11 weeks	Aspirate & Tissue: (+) SBG	27,248 cels, 97% PMN	16 yrs
Mean		44.4% with prior revision	30 weeks of symptoms (0.3 - 175)	100% aspirate (+) SBG 55.5% Tissue (+) SBG 22.2% additional pathogen	89,586.3 Cells (10,625 - 321,380) 95.9% PMN (85% - 98%)	8 yrs (4 weeks - 17 years)
